# CEP19 cooperates with FOP and CEP350 to drive early steps in the ciliogenesis programme

**DOI:** 10.1098/rsob.170114

**Published:** 2017-06-28

**Authors:** Bahareh A. Mojarad, Gagan D. Gupta, Monica Hasegan, Oumou Goudiam, Renata Basto, Anne-Claude Gingras, Laurence Pelletier

**Affiliations:** 1 Lunenfeld-Tanenbaum Research Institute, Mount Sinai Hospital, 600 University Avenue, Toronto, Ontario, CanadaM5G 1X5; 2 Department of Molecular Genetics, University of Toronto, Toronto, Ontario, CanadaM5S 1A8; 3 Institut Curie, PSL Research University, CNRS, UMR144, 12 rue Lhomond, Paris 75005, France

**Keywords:** centrosomes, cilia, centrioles, ciliopathies, super-resolution microscopy

## Abstract

Primary cilia are microtubule-based sensory organelles necessary for efficient transduction of extracellular cues. To initiate cilia formation, ciliary vesicles (CVs) are transported to the vicinity of the centrosome where they dock to the distal end of the mother centriole and fuse to initiate cilium assembly. However, to this date, the early steps in cilia formation remain incompletely understood. Here, we demonstrate functional interplay between CEP19, FOP and CEP350 in ciliogenesis. Using three-dimensional structured-illumination microscopy (3D-SIM) imaging, we mapped the relative spatial distribution of these proteins at the distal end of the mother centriole and show that CEP350/FOP act upstream of CEP19 in their recruitment hierarchy. We demonstrate that CEP19 CRISPR KO cells are severely impaired in their ability to form cilia, analogous to the loss of function of CEP19 binding partners FOP and CEP350. Notably, in the absence of CEP19 microtubule anchoring at centromes is similar in manner to its interaction partners FOP and CEP350. Using GFP-tagged deletion constructs of CEP19, we show that the C-terminus of CEP19 is required for both its localization to centrioles and for its function in ciliogenesis. Critically, this region also mediates the interaction between CEP19 and FOP/CEP350. Interestingly, a morbid-obesity-associated R82* truncated mutant of CEP19 cannot ciliate nor interact with FOP and CEP350, indicative of a putative role for CEP19 in ciliopathies. Finally, analysis of CEP19 KO cells using thin-section electron microscopy revealed marked defects in the docking of CVs to the distal end of the mother centrioles. Together, these data demonstrate a role for the CEP19, FOP and CEP350 module in ciliogenesis and the possible effect of disrupting their functions in ciliopathies.

## 1. Introduction

The centrosome is the major microtubule (MT) organizing centre in animal cells and consists of two centrioles embedded in pericentriolar material [1]. The two centrioles are structurally and functionally distinct, with the older, so-called mother centriole harbouring appendage proteins at its distal end. Based on their localization along the mother centriole, appendage proteins are either classified as distal appendage proteins (DAPs) or subdistal appendage proteins (sDAPs). The centrosome is essential for the formation of primary cilia [2,3], MT-based organelles that function as the cell's antenna [3]. When the cell exits the cell cycle and enters a quiescent state (G0 phase), the centrosome moves to the vicinity of the plasma membrane where the mother centriole transitions to a basal body state, templating the formation of the cilium, by extending MT doublets to form the ciliary axoneme [4]. The ciliogenesis process begins with the migration of ciliary vesicles (CVs) to the centriole vicinity, followed by their docking to the distal end of the mother centrioles [5]. Subsequently, CP110, a distal end centriole protein, gets removed from the distal end of the mother centriole only [6], and the ciliary axoneme is extended. This step is followed by the loading of Bardet–Biedl syndrome (BBS) proteins, which in turn assemble intraflagellar transport (IFT) complexes required for cargo transport in the cilium [5,7]. Mutations affecting the formation or function of cilia are associated with genetic disorders collectively called ciliopathies. Ciliopathies are characterized by retinal degeneration, renal disease, cerebral anomalies, diabetes, obesity and skeletal dysplasias [8]. Several centriolar appendage proteins, including CEP164, SCLT1 and CEP83, which play a key role in the process of ciliogenesis by docking of the CVs to the mother centriole, are mutated in ciliopathies [9–11]. CEP19 (C3ORF34) preferentially localizes to the mother centriole [12] and GFP-CEP19 co-localizes with NIN at the subdistal appendages [13]; however, the role of CEP19 in centrosome structure and function is unknown. Here, we show that CEP19 interacts with two centrosomal proteins, FGFR1OP (FOP) and CEP350, and is essential for the recruitment of CVs to the mother centriole and, consequently, for ciliogenesis. CEP19 truncations that disrupt its interactions with FOP and CEP350, including a truncation mutation that is associated with morbid obesity syndrome, prevent ciliogenesis [14].

## 2. Results and discussion

### 2.1. CEP19 interacts with and requires FOP and CEP350 for localization to the centrosome

**Figure 1.**
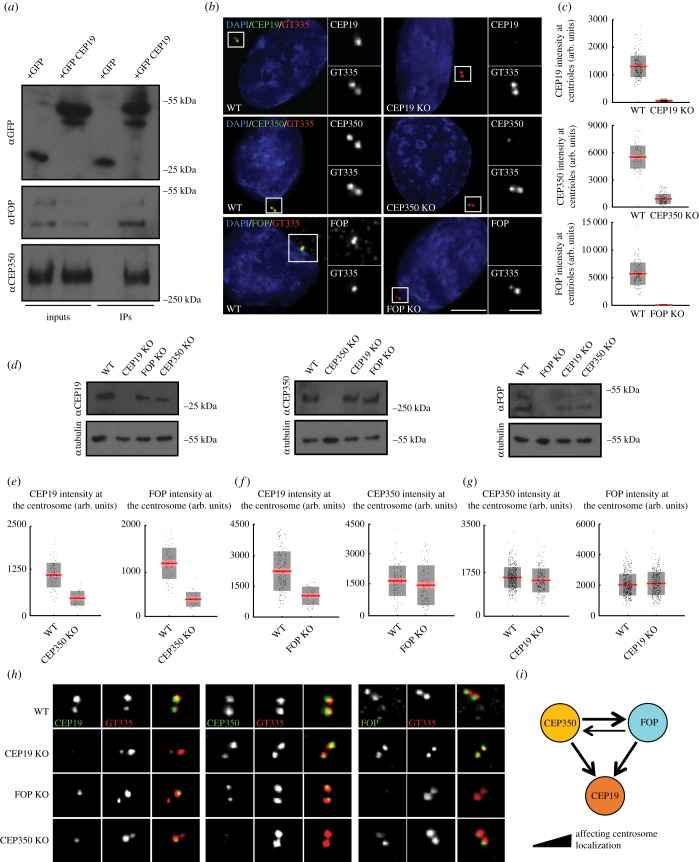
CEP19, FOP and CEP350 interact and localize to the distal end of centrioles. (*a*) GFP-CEP19 interacts with endogenous FOP and CEP350. (*b*) Generation of CRISPR KO RPE-1 cell lines for CEP19, FOP and CEP350. IF micrographs of WT and knockout RPE-1 cells probed with the indicated antibodies and counterstained with DAPI. Scale bar, 5 µm. Insets 1 µm. (*c*) Mean fluorescence intensity of the indicated proteins at the centrosome in WT, CEP19, CEP350 and FOP KO RPE-1 cell lines. Grey region denotes 2 s.d. from the mean (red line), pink region denotes 95% confidence interval. (*d*) Western blots confirming the loss of expression of the indicated proteins. (*e*) Quantification of the mean fluorescence intensity of the indicated protein in CEP350 KO RPE-1 cell line. (*f*) Quantification of the mean fluorescence intensity of the indicated protein in FOP KO RPE-1 cell line. (*g*) Quantification of the mean fluorescence intensity of the indicated protein in CEP19 KO RPE-1 cell line. (*h*) CEP19 localization to the centrioles is dependent on FOP and CEP350. (*i*) Assembly pathway of CEP19, FOP and CEP350.

In our previous large-scale study of the *in vivo* proximity interactors of centrosomal proteins in HEK293 cells [13], the most abundant high-confidence proximity interactors for CEP19 included two known centrosomal proteins: FGFR1OP (FOP) and its interaction partner CEP350. CEP19 was also identified as an interaction partner for FOP using affinity purification coupled to mass spectrometry (AP-MS) and yeast-two hybrid [15,16]. Furthermore, FOP was previously shown to directly interact with the C-terminal domain of CEP350, and both proteins are required for MT anchoring at the centrosome [17]. To further determine whether the proximity interactions between CEP19, FOP and CEP350 result in the formation of biochemically stable interactions, CEP19 was fused to a FLAG tag and used for AP-MS. In this context, both FOP and CEP350 were found to be high-confidence interactors for CEP19, with FOP detected with the highest spectral counts (electronic supplementary material, figure S1*a*; table S1). The interactions between GFP-tagged CEP19 and endogenous FOP and CEP350 were confirmed using co-immunoprecipitation (Co-IP) experiments in HEK293 cells (figure 1*a*). These three independent lines of evidence support the notion that CEP19, FOP and CEP350 associate *in vivo*.

### 2.2. CEP350 and FOP are required for robust recruitment of CEP19 to centrosomes

The interaction data detailed above led us to hypothesize that CEP350, FOP and CEP19 would rely on each other for their localization to centrosomes. To investigate the recruitment dependency of these proteins, we set out to examine the localization of each protein in cells where the expression of the others was perturbed. We generated CRISPR knockout cell lines for each protein and confirmed the loss of expression by quantitative immunofluorescence microscopy (figure 1*b,c*) and western blot analysis using antibodies against the endogenous proteins (figure 1*d*). We found that in CEP350 KO cells, the recruitment of FOP and CEP19 to the centrioles was markedly decreased (figure 1*e*,*h*). Similarly, loss of FOP disrupted the recruitment of CEP19 and partially affected CEP350 localization to the centrioles (figure 1*f*,*h*). However, centriolar localization of FOP and CEP350 remained largely intact in the absence of CEP19 (figure 1*g*,*h*). We did note a small decrease in total FOP levels in CEP19 and CEP350 KOs, suggestive of a potential role for these two proteins in stabilizing FOP. Together, these data indicate that FOP and CEP350 are both required for the efficient localization of CEP19 to the centrosome, but not vice versa (figure 1*i*).

We had previously shown using 3D-SIM [13] that GFP-CEP19 localized in the vicinity of the sDAP NIN. We therefore sought to examine whether the absence of CEP19 affects the recruitment of DAPs or sDAPs in RPE-1 cells (electronic supplementary material, figure S1*b*). We observed that CEP19 removal only partially affected the recruitment of the sDAPs CEP170 and NIN, but did not affect the recruitment of the DAP CEP164 (electronic supplementary material, figure S1*b*,*c*). We also examined the recruitment of CEP19 in cells depleted of the sDAPs ODF2, NIN, CEP170 and CEP128, and the DAPs CEP164, CEP83 and CEP89; however, none of these proteins significantly affected the recruitment of CEP19 to centrioles (electronic supplementary material, figure S2*a*,*b*). Together, these results indicate that the localization of the CEP19/CEP350/FOP module is largely independent of other sDAP and DAP components.

### 2.3. Subdiffraction profiling of CEP19, FOP and CEP350 at centrioles

**Figure 2.**
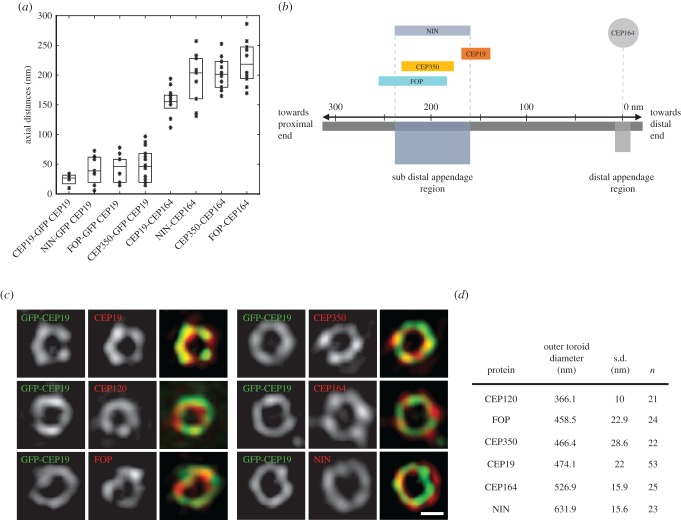
Three-dimensional SIM-based localization of CEP19, FOP and CEP350 at the distal end of the centrioles. (*a*) Axial distance of indicated proteins with respect to GFP-CEP19, or CEP164 (the median together with the 25% and 75% quantiles are shown for each category; *n* > 10). (*b*) Schematic overview of relative localization of indicated proteins along the length of the centriole in HeLa cells. (*c*) Three-dimensional SIM micrographs of centrioles immuno-labelled with GFP-CEP19 (in green, imaged at 488 nm), indicated centriolar proteins (in red, imaged at either 568 or 642 nm) and merged channels. Scale bar, 250 nm. (*d*) Table summarizing the outer ring diameter of the indicated proteins.

We next used 3D-SIM to examine the precise localization of CEP19, FOP and CEP350 with respect to other centriole proteins. To do this, we used a method we previously developed to quantify the axial and toroidal distribution of centriole components with high precision [13,18]. To detect these proteins, we used antibodies specific to endogenous FOP and CEP350 in combination with anti-GFP labelling of a stable cell line expressing N-terminally tagged GFP-CEP19. NIN and CEP164 labelling was used to demarcate the sDAP and DAP domains at the distal end of centrioles, respectively [19] (figure 2). We screened hundreds of centrioles and manually selected only those that were oriented perpendicular to the imaging plane for measurements (see Material and methods for details). Three-dimensional quantitative analysis of axial line profiles indicated that FOP and CEP350 were both approximately 40–45 nm away from CEP19, and both CEP350 and FOP reside approximately 50–70 nm more proximally than CEP19, which is closer to the DAP protein CEP164 (see figure 2*a* and 2*b*, for measurements and relative spatial distribution on the centriole, respectively). Furthermore, in transverse sections, FOP, CEP350 and CEP19 antigens were localized as rings at the distal end of centrioles (figure 2*c*,*d*). All three antigens displayed a very similar ring diameter (approx. 465 nm) as measured in maximum *z*-projections. This was significantly different from CEP164 and NIN, distal and subdistal appendage markers, and CEP120, a centriole proximal marker (figure 2*c*,*d*) [20]. Together, these results indicate a close association of these proteins at the distal end of centrioles, with their relative localization spanning the sDAP region.

### 2.4. CEP19, FOP and CEP350 localize to the basal body in ciliated cells and are required for ciliogenesis

**Figure 3.**
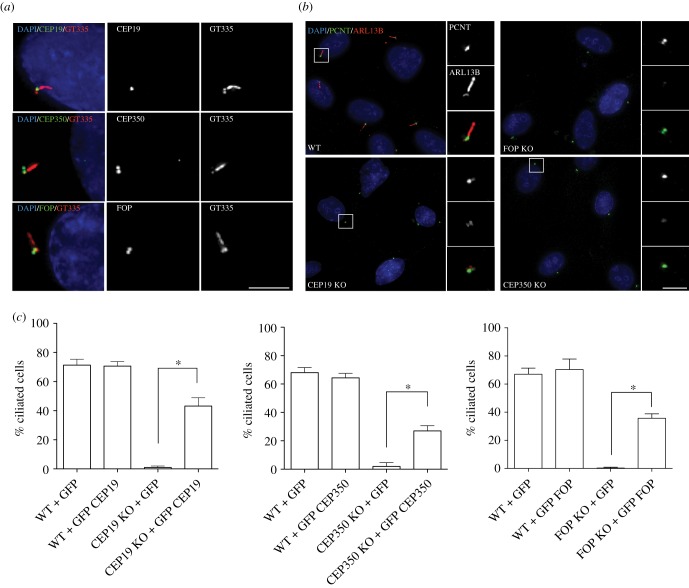
CEP19, FOP and CEP350 are required for ciliogenesis. (*a*) CEP19, FOP and CEP350 localize to the basal body in serum-starved WT RPE-1 cells. Scale bar, 5 µm. (*b*) IF micrographs of CEP19, FOP and CEP350 KO and WT RPE-1 cell lines upon serum starvation. Main merged panels are immuno-labelled with PCNT (green), ARL13B (red) and counterstained with DAPI (blue), corresponding top and bottom insets represent PCNT and ARL13B, respectively. Scale bar, 15 µm. Insets 1 µm. (*c*) Expression of GFP-tagged CEP19, FOP and CEP350 rescues the defect in ciliogenesis in their respective cell lines. *p < 0.01 by Student's *t*-test, *n* > 200.

The CEP19 interactor, FOP, was previously shown to be a positive regulator of ciliogenesis [21], and the localization of the CEP19/CEP350/FOP module at the distal end of the mother centriole is suggestive of a role for CEP19 and CEP350 in ciliogenesis. To test this possibility, we first examined the localization of these proteins in ciliated cells. RPE-1 cells were serum-starved for 72 h to induce ciliogenesis, fixed and labelled for GT335 as a marker of cilia, and for CEP19, FOP or CEP350. We observed that CEP19, FOP and CEP350 all localized to the base of the cilium in these cells (figure 3*a*). When CEP19, CEP350 and FOP CRISPR KO cell lines were similarly serum-starved, ciliogenesis was strongly perturbed compared with isogenic WT RPE-1 cells (figure 3*b*,*c*). The defects in ciliogenesis observed in CEP19, FOP and CEP350 KO cells could be robustly rescued through the transient expression of GFP-tagged variants of the corresponding protein (figure 3*c*; electronic supplementary material, figure S2*c*). In the case of CEP19, these results could be reproduced using CEP19-specific siRNAs (electronic supplementary material, figure S3*a*,*b*). Taking into account the role of CEP350 and FOP in MT anchoring [17], and the role for other sDAPs like NIN in MT anchoring [22], we next investigated the role of the CEP19/CEP350/FOP module in this process. Using classical regrowth experiments, in combination with imaging of the tubulin network, robust defects in MT anchoring could be observed following 20 min of regrowth for CEP350 and FOP RNAi-treated cells (electronic supplementary material, figure S3*c*,*d*). A less severe, though significant defect was also observed in siCEP19-treated cells (electronic supplementary material, figure S3*c*,*d*). Together these data suggest that perturbing the function of the CEP19/CEP350/FOP module impairs ciliogenesis, and the ability of cells to anchor MTs at centrosomes.

### 2.5. Structure–function analysis of the CEP19/CEP350/FOP module

**Figure 4.**
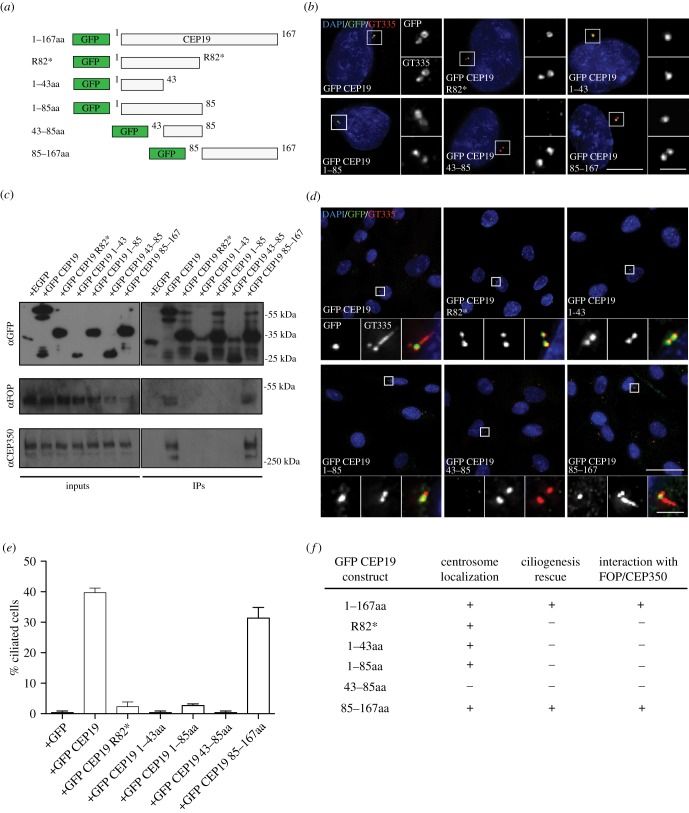
Mapping the centriole localization and ciliogenesis domain in CEP19. (*a*) Schematics of various GFP-tagged truncation fragments generated for CEP19. (*b*) IF micrographs representing the localization of GFP-tagged CEP19 deletion fragments in cycling WT RPE-1 cells. Main merged panels are immuno-labelled with GFP (green), GT335 (red) and counterstained with DAPI (blue), corresponding top and bottom insets represent GFP and GT335, respectively. Scale bar, 5 µm. Insets 1 µm. (*c*) Co-IPs showing the interaction of GFP-tagged CEP19 fragments with endogenous FOP and CEP350. (*d*) IF micrographs of expressed GFP-tagged CEP19 deletion fragments in serum-starved CEP19 KO cells to monitor ciliogenesis. Main merged panels are labelled with GFP (green), GT335 (red) and counterstained with DAPI (blue), corresponding left and middle insets represent GFP and GT335, respectively. Scale bar, 15 µm. Insets 1 µm. (*e*) Quantifications of percentage of ciliated cells in the presence of indicated GFP-tagged deletion fragments in serum-starved RPE-1 cells. (*f*) Summary of different CEP19 fragments generated and their ability to rescue ciliogenesis in CEP19 KO cells and interact with FOP and CEP350.

CEP19 is a small protein of 167 amino acids with no identified domains. In order to determine which part of CEP19 is required to target the protein to the centrioles, we made deletion mutants of CEP19, while preserving its protein secondary structure (figure 4*a*; electronic supplementary material, figure S3*e*). We also generated an altered form of CEP19 with a nonsense homozygous mutation at codon 82 of exon 2 (R82*). The R82* mutation, which results in a truncated protein that lacks the C-terminal domain, was found in multiple family members in a linkage analysis study of an autosomal recessive Mendelian disorder [14].

We first examined the ability of each deletion construct to localize to the centrosome. RPE-1 cells were transiently transfected with the GFP-CEP19 deletion constructs and processed for IF with GFP antibodies to examine their subcellular localization. Truncated forms carrying the N-terminal 43 residues (1–43) and the C-terminal half of the protein (residues 85–167), as well as the R82* mutant form, were able to localize to the centrioles, while other truncations were not (figure 4*b*).

We next examined the ability of CEP19 N- and C-terminal deletions to rescue ciliation defects observed in CEP19 KO cells, by expressing their GFP-tagged variants in serum-starved CEP19 KO RPE-1 cells. We found that the C-terminal half of CEP19 was able to restore ciliation (figure 4*d*), while other truncations, including the R82* mutant, were unable to do so (figure 4*d*,*e*). Furthermore, we examined the ability of CEP19 fragments to interact with FOP and CEP350. Our results show that both the full-length protein and its C-terminal half could interact with FOP and CEP350, whereas the other truncated mutants, including the R82* mutant, did not (figure 4*c*). Overall, these results show that CEP19's C-terminal region plays a critical role in ciliogenesis. Specifically, this region is required for its robust association with FOP/CEP350 and localization to centrosomes. Importantly, this suggests that the obesity-associated R82* mutation disrupts CEP19's interaction with FOP and CEP350, thereby resulting in defective ciliogenesis [14].

### 2.6. CEP19 is required for the early steps of ciliogenesis

**Figure 5.**
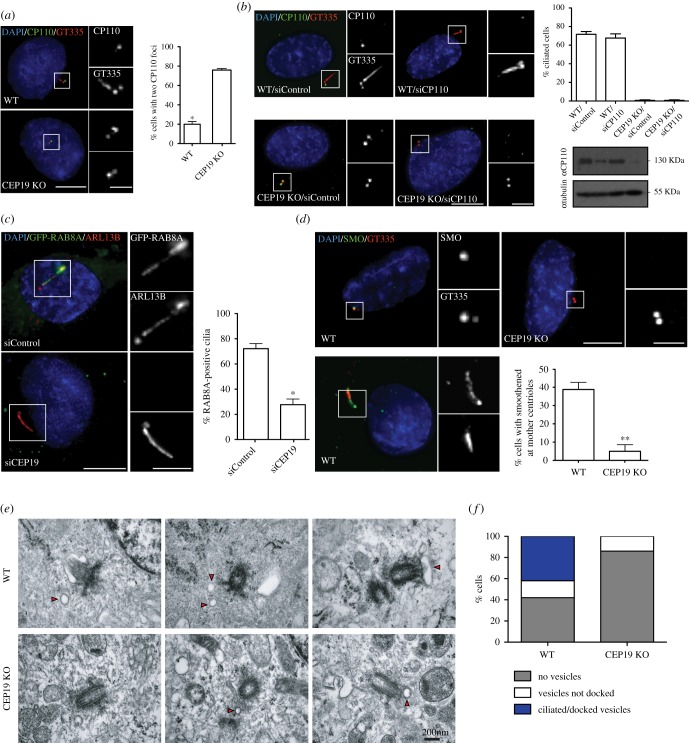
CEP19 is required for the early stages of ciliogenesis. (*a*) IF micrographs of serum-starved WT and CEP19 KO RPE-1 cells. Main merged panels are immuno-labelled with CP110 (green), GT335 (red) and counterstained with DAPI (blue), corresponding top and bottom insets represent CP110 and GT335, respectively. Graph shows the percentage of cells with two CP110 foci at the centrioles in serum-starved WT and CEP19 KO RPE-1 cells. Scale bar, 5 µm. Insets 1 µm. *p < 0.01 by Student's *t*-test, *n* > 200. (*b*) IF micrographs of serum-starved WT or CEP19 KO RPE-1 cells treated with non-targeting siRNA or siRNA targeting CP110. Main merged panels are immuno-labelled with CP110 (green), GT335 (red) and counterstained with DAPI (blue), corresponding top and bottom insets represent CP110 and GT335, respectively. Scale bar, 5 µm. Insets 1 µm. Top right: quantification of percentage of ciliated cells in control or CEP19 KO cells, with siControl or siCP110. Bottom right: western blot (in the same row order as graph) confirming the depletion of CP110. (*c*) IF micrographs of GFP-RAB8A RPE-1 cells serum-starved for 24 h. Main merged panels are immuno-labelled with GFP (green), ARL13B (red) and counterstained with DAPI (blue), corresponding top and bottom insets represent GFP and ARL13B, respectively. Graph shows percentage of GFP-RAB8A positive cilia in controls compared with CEP19-depleted RPE-1 cells. Scale bar, 5 µm. Insets 1 µm. (*d*) IF micrographs of serum-starved WT RPE-1 and CEP19 KO RPE-1 cells treated with SAG. Main merged panels are immuno-labelled with SMO (green), GT335 (red) and counterstained with DAPI (blue), corresponding top and bottom insets represent SMO and GT335, respectively. Graph shows percentage of cells with smoothened at the mother centriole in WT compared with CEP19 KO cells. Scale bar, 5 µm. Insets 1 µm. (*e*) Electron micrographs showing serial sections of WT and CEP19 KO RPE-1 cells serum-starved for 72 h. Arrowheads indicate CVs. (*f*) Quantification of percentage of cells with docked/undocked CVs and no vesicles in WT and CEP19 KO RPE-1 cells.

In order to pinpoint CEP19's function in ciliogenesis, we examined the status of different steps of the ciliogenesis programme in CEP19 KO cells. We first examined if removal of CP110 from the distal end of the mother centriole (a key step in the ciliogenesis pathway [23]) occurred normally in the absence of CEP19. CEP19 KO cells were serum-starved for 72 h and labelled with CP110, along with GT335 as a cilia marker. Eighty per cent of cells in the CEP19 KO cell line displayed CP110 foci at both mother and daughter centrioles, whereas only 20% of cells in WT had two CP110 foci at the distal end of both their centrioles, with the majority (approx. 80%) of WT cells being able to ciliate (figure 5*a*). Subsequently, we examined whether bypassing CP110 removal in CEP19 KO cells could rescue the defective ciliation. CP110 was depleted from CEP19 KO cells using CP110-specific siRNAs, and the cells were serum-starved for 72 h. Robust depletion of CP110 was achieved as judged by western blot (figure 5*b*). This resulted in an approximately 70% drop in the percentage of cells with strong CP110 labelling in CEP19 KO cells (figure 5*b*). Nonetheless, removal of CP110 from the mother centriole was not sufficient to rescue the defective ciliation in these cells, suggesting that the defect in the ciliogenesis process is occurring at an earlier step, prior to CP110 removal (figure 5*b*). Similar results were obtained using CEP19-specific siRNAs (electronic supplementary material, figure S4*a*).

Consequently, we next examined the requirement for CEP19 in recruiting RAB8A to the ciliary membrane [24]. RAB8A is a positive regulator of ciliogenesis, which associates with the basal body and is required for ciliary membrane assembly [25]. As the number of ciliated cells in CEP19 KO cells was too low to analyse, we used CEP19 siRNA-treated RPE-1 cells that manage to ciliate, albeit at a lower frequency. Cilia in serum-starved WT RPE-1 cells displayed 70% association of GFP-RAB8A, whereas in CEP19 siRNA-depleted cells, only 30% of cilia showed RAB8A association indicative of a defect in RAB8A recruitment (figure 5*c*). The recruitment of Smoothened (SMO), a CV marker [26], to the distal end of the mother centriole was also monitored. Cells serum-starved for 72 h were treated with SAG (smoothened agonist) and labelled with SMO antibody. CEP19 KO cells displayed an approximately 30% decrease in the number of SMO-associated non-ciliated centrioles compared with controls (figure 5*d*). Defects in IFT loading, using IFT88 as a marker, were also observed (electronic supplementary material, figure S4*b*), suggesting that CEP19 may act as early as CV formation and/or docking [27].

A survey of 35 serum-starved WT RPE-1 cells using electron microscopy revealed that CVs were largely within the vicinity of the centrosomes and/or docked to the distal end of the centrioles. By contrast, in 42 centrioles analysed in CEP19 KO cells, we were unable to find any CVs docked to the distal end, even though we could detect CVs in the centrosome vicinity (figure 5*e*,*f*). Together, these results indicate that in the absence of CEP19, CVs are not able to dock to the mother centrioles, suggesting that CEP19 is required for initiation of ciliogenesis.

## 3. Discussion

Using a combination of RNAi, CRISPR KO and Co-Ip studies, we describe a role for CEP19 in ciliogenesis, which is dependent upon its ability to interact with FOP and CEP350. We find that FOP and CEP350 facilitate the localization of CEP19 to the centriole, but a number of DAPs (CEP83, CEP89 and CEP164) or sDAPs (CEP128, CEP170, NIN and ODF2) were not required for centriolar targeting of CEP19. Furthermore, 3D-SIM analysis indicates that CEP19, FOP and CEP350 localize to within approximately 200 nm from the distal end of centrioles, consistent with their interaction. The recruitment dependency of these three proteins at centrioles, their colocalization at the distal end, and their interaction and mutual requirement for ciliogenesis suggest that these proteins are acting as a functional module to positively regulate cilia formation.

CEP19 KO mice are morbidly obese, glucose intolerant and resistant to insulin. In mice and humans, the CEP19 R82* nonsense mutation is associated with morbid obesity [14]. The inability of CEP19 R82* to restore ciliation in CEP19 KO cells may point to a ciliopathic explanation of how this homozygous mutation is linked to obesity. Several ciliopathy disorders (including BBS and Alström syndrome) are also associated with obesity and diabetes [28]. As defects in BBS proteins are associated with obesity [29], and CEP19 R82* mutant patients are morbidly obese [14], it is possible that CEP19 cooperates with BBS proteins to form cilia. Further studies will be required to explore the ability of CEP19, as well as the R82* mutant, to interact with BBS proteins and/or cargo to decipher the role of CEP19 in these diseases. Individuals carrying the homozygous nonsense mutation in the CEP19 gene studied in [14] were affected by morbid obesity, T2DM, heart defects and hypertension. Furthermore, three out of five individuals with early coronary artery disease died as a result of cardiac defects. Twenty-seven per cent of individuals had intellectual disability and all six males studied had decreased sperm counts. These conditions fall within the spectrum of diseases associated with ciliopathies, which can vary in penetrance [30]. Importantly, our study was done in human RPE-1 cells that are retinal epithelial cells. It would be interesting to investigate the role of CEP19 in various tissue and organoid models, in particular the ones that are directly affected in ciliopathies, like kidney and liver.

CEP19-depleted cells retain CP110 at the distal end of the mother centrioles, suggesting that CEP19 is required for an early step during ciliogenesis. Furthermore, these cells are unable to efficiently recruit Smoothened, a membrane protein recruited quickly upon CV formation, to the basal body. EM analysis of mother centrioles from serum-starved CEP19 KO cells displayed a marked absence of docked CVs. Together, these data suggest that CEP19 might be regulating CV docking during early ciliogenesis. Therefore, it will be interesting to investigate a possible link/interaction between CEP19 and early ciliary membrane components. Potential candidates include the membrane-shaping EHD1/EHD3 proteins that cooperate with Rab11 and Rab8 in early in ciliogenesis, at the stage of CV formation and IFT loading [7,31]. Another plausible candidate could be centriolar satellites, approximately 70 nm electron-dense granules present in the vicinity of the centrosomes and the cell periphery that have been implicated in different processes, including ciliogenesis [21,32,33]. This possibility is supported by our observation that CEP19 associates with PCM1, a major component of centriolar satellites [34] (electronic supplementary material, table S1), and recent reports that satellite components WRAP73 and SSX2IP function together to drive CV formation [13,35,36]. It was also recently shown that CEP19 associates with, and is required to recruit, the Rab protein RABL2 to the basal body, which can promote cilia assembly via its interaction with the IFT-B complex [37]. Consistent with this observation, we also identified a putative interaction between FLAG-CEP19 and RABL2A in our AP-MS data (electronic supplementary material, table S1). As we also observed defects in IFT88 and Rab8 recruitment in CEP19 KO cells, this may suggest that CEP19 could be an important regulator for membrane trafficking pathways at the basal body during ciliogenesis. Through monitoring of the MT network upon recovery from cold treatment, we noted marked defects in MT anchoring in CEP350 and FOP KO cells, and to a lesser degree in CEP19 KO cells. This is consistent with previously established roles of CEP350 and FOP in MT anchoring [17]. CEP19 displays a less dramatic effect on MT anchoring indicative of a more ancillary role in this process. FOP had previously been shown to be required for ciliogenesis [21], and our results extend this requirement to the two other members of this regulatory module—CEP350 and CEP19. The fact that depleting any component of this module leads to defects in both ciliation and anchoring raises the possibility that MT anchoring may be required for the ciliogenesis programme, perhaps via the efficient targeting of CVs through MT-dependent vesicular transport.

Taken together, the work presented here defines a novel function for the CEP19/CEP350/FOP module at the distal end of the centriole to drive early steps in ciliogenesis.

## 4. Material and methods

### 4.1. Cell lines

HEK 293 cells (Invitrogen) and HeLa cells were grown in Dulbecco's Modified Eagle's Medium (DMEM) supplemented with 10% fetal bovine serum (FBS), and GlutaMAX. hTERT RPE-1 cells were grown in DMEM/F12 supplemented with 10% FBS, GlutaMAX and sodium bicarbonate (1.2 g l^−1^).

### 4.2. CRISPR-mediated gene disruption

pX458-CEP19, CEP350 and FOP were created by cloning annealed oligos corresponding to exons 2 and 3 of CEP19, exon 1 of FOP and exons 2 and 3 of CEP350 into pX458-U6-Chimeric_BB-CBh-hSpCas9 (**ADDGENE reference 42230). A total of 40 000 hTERT RPE-1 cells were seeded in a 24-well plate and transfected with 0.5 µg targeting plasmid with Lipofectamine 3000 (Invitrogen). Twenty-four hours post-transfection, single clones were obtained using fluorescence-activated cell sorting to sort GFP-positive cells. Gene disruption was confirmed by directly sequencing PCR amplicons of the targeted region, by sequencing individually cloned PCR amplicons and western blot and immunofluorescence analysis of CEP19, FOP and CEP350.

### 4.3. Antibodies

See electronic supplementary material, table S2 for information about antibodies used in this study. In the CEP350 KO cells we generated in this study, the quantified CEP350 signal at centrioles by IF is not completely abolished. This contrasts with CEP19 and FOP that is virtually undetectable in KO cells. By western blot, the CEP350 KO cells seem to be devoid of CEP350. We thus speculate that the residual CEP350 signal at centrioles (approx. 5–10% of total) in the immunofluorescence images could be due to the cross-reactivity of CEP350 antibody with other centrosomal proteins. The use of a polyclonal antibody for CEP350 in this study might have contributed to its possible cross-reactivity with other proteins. Indeed, CEP350 has 40% sequence similarity to DCTN1, a known centriolar protein, which may explain the low level of cross-reactivity of the polyclonal CEP350 antibody used in this study. Here, we used Rabbit polyclonal FOP antibody (Proteintech-11343-1-AP) and we detected a doublet band for endogenous FOP. Detecting a doublet band for FOP was also reported using other antibodies, for example, the mouse monoclonal FOP antibody (Abnova- H00011116-M02) was used by Lee & Stearns [21] and two bands were detected for FOP. Similarly, the FOP antibody from Bethyl (A301-860A) detects a doublet band for endogenous FOP. FOP has three isoforms, the longest of which has 399 amino acids and is approximately 43 kDa and an alternately spliced variant of 351aa and a predicted molecular weight of 38 kDa. The latter could be the band migrating at a lower weight on SDS–PAGE.

### 4.4. Ciliogenesis experiments

hTERT RPE-1cells were reverse transfected with Dharmacon ON-TARGETplus siRNA deconvolved pool on 6-well plates in complete medium. Twenty-four hours after transfection, the cells were starved in serum-free medium for 48 h after which they were fixed in ice-cold methanol and immune-stained with antibodies directed against the protein-of-interest and GT335/ARL13B as cilia markers. Images were acquired on DeltaVision microscopes. Three-dimensional datasets were acquired for up to 300 cells per condition. Levels of ciliogenesis were manually calculated using GT335/ARL13B channel for cilia. Ciliation in cells transfected with non-targeting siRNA was used as control.

### 4.5. Super-resolution microscopy

Super-resolution imaging was performed as described previously [20]. Briefly, cells were imaged on a 3D-SIM (OMX Blaze v4, GE Biosciences PA) equipped with 405, 445, 488, 514, 568 and 642 nm diode lasers, four high-speed sCMOS cameras (scientific CMOS, 2560 × 2560 pixels^2^, manufactured by PCO), and a ×60/1.42 NA planApochromat oil-immersion objective (Olympus). Multi-channel 3D-SIM image Z stacks (25 sections, 0.125 µm apart) were reconstructed, three-dimensional aligned using calibrations based on a GE reference slide and 100 nm diameter TetraSpeck Microspheres and maximum intensity projected using the softWoRx 6.0 software package (GE). The 3D-SIM modality of our Optical Microscopy eXperiment (OMX) provides an axial resolution of 340–380 nm depending on the imaging wavelength, thus sampling axially every 125 nm satisfied the Nyquist criterion for oversampling (GE Biosciences PA).

### 4.6. RNA interference

All siRNA transfections were performed using the Lipofectamine RNAiMAX transfection reagent (Invitrogen) according to the manufacturer's instructions. The non-targeting siRNA from Dharmacon was used as a negative control. To silence CEP19, RPE-1 cells (2 × 10^5^ cells seeded in 6-well plates) were transfected with 40 nM (final concentration) of one Dharmacon ON-TARGETplus siRNA targeting CEP19. For co-depletion, CEP19 and CP110 were initially transfected with the negative control siRNA or the CEP19 siRNA deconvolved. Twenty-four hours post-transfection, the cells were transfected with either the negative control siRNA or CP110 siRNA pool for an additional 48 h during which the cells were serum-starved.

### 4.7. RNAi rescue experiments

To rescue the CEP19 phenotype, hTERT RPE-1 cells were transfected with 40 nM CEP19 siRNA. Twenty-four hours post-transfection, cells were transfected with 1 µg of siRNA-resistant GFP-CEP19 (GCAAGATCCGGCAGCGGAT) using Lipofectamine 3000 (Invitrogen). This allowed for the knockdown of endogenous CEP19 but not siRNA-resistant GFP-CEP19. After 6 h, cells were serum-starved for 48 h and then fixed and processed for immunofluorescence analysis. Efficient depletion of endogenous CEP19 was confirmed by western blot analysis.

### 4.8. Image analysis: line profiles

For line profile analysis, we used the same method as in [18]. Briefly, 3D-SIM, three-channel, *z*-stack images of protein-of-interests were taken in 488, 568 and 642 nm channels, and then reconstructed and aligned in softWoRx. The 3D-SIM modality of our OMX provides an axial resolution of 340–380 nm depending on the imaging wavelength, thus sampling axially every 125 nm satisfied the Nyquist criterion for oversampling (GE). For each imaging channel, all 16-bit *z*-sections were manually chained in a montage strip in ImageJ. For each triplet of images, line profiles across montages were drawn at the same position and the intensity profiles were recorded (in ImageJ). The profiles were normalized to their respective maximum intensity, and the peaks brighter than 30% of the maximum intensity were fit with a one-dimensional Gaussian function (in MATLAB). The distance along axial axis between two proteins was calculated as the difference between their corresponding Gaussian peaks. Figure 2*a* presents averages of such measurements between various protein-of-interest with respect to CEP19 and CEP164, as well as of control measurements between CEP19 imaged in two channels at the same time (GFP-CEP19 in 448 and CEP19 in 568 nm). Note that these distances are dependent on several factors with this technique and are provided as relative measurements, including: the axial resolution, the epitopal configuration of the protein being detected, the length of the appendage structure and the angle at which the appendage protrudes from the mother centriole.

### 4.9. Image analysis: diameter measurements

Measurements of diameters of protein-of-interests in interphase were derived following the same procedure described in [18]. Briefly, we used our in-house developed MATLAB routine to interactively select the boundary pixels of centrosome XY rings in 16-bit softWoRx reconstructed, aligned and projected 3D-SIM images in one of the 488, 568 and 642 nm channels. The diameter of the fitted circle to the boundary pixels provided the protein size in *xy* dimensions. Figure 2*d* shows average measurements for protein-of-interests.

### 4.10. Image analysis: protein recruitment and quantification at centrioles or centrosomes

To quantify centriole or centrosome recruitment of various antigens, six *z*-planes of 1.5 µm and greater than 300 cells per sample were acquired at 60×/1.4NA (2× binning) on a Deltavision Elite DV with a 2048 × 2048 sCMOS camera (GE Life Sciences, PA). Image analysis employing adaptive thresholding was carried out on deconvolved, *z*-projected stacks with MATLAB to identify centrioles or centrosomes using specific markers as specified in the text (see also [13]). The resulting centriole mask was dilated by 3 pixels radially and then applied to dark-noise subtracted original images to obtain the mean and integrated pixel intensity for each channel, for every cell.

### 4.11. Electron microscopy

For thin-section EM, CEP19 K hTERT RPE-1 cells were grown in 10 cm plates in either complete medium or serum-starved for 72 h, fixed for 1 h in 2% glutaraldehyde in sodium cacodylate buffer for 1 h, and 24 h at 4°C and post-fixed in 1% osmium tetroxide. Samples were dehydrated through a graded series of ethanol followed by propylene oxide and embedded in Embed 812 resin. Thin sections (90 nm) were cut on an RMC MT6000 ultramicrotome, stained with 2% uranyl acetate in 70% methanol and then aqueous lead citrate. Samples were viewed on FEI Tecnai 20 transmission electron microscope.

### 4.12. Western blots

For western blots, cells were collected, lysed in Laemmli buffer and treated with benzonase nuclease (Sigma-Aldrich). Proteins were separated by loading whole-cell lysates onto a 6–12% SDS–PAGE gel for electrophoresis and then transferred to a PVDF membrane (Immobilon-P, Millipore). Membranes were incubated with primary antibodies in TBST (TBS, 0.1% Tween-20) in 5% skim milk powder (BioShop), supplemented with 2.5% BSA Fraction V (OmniPur) in the case of FOP western blots. Blots were washed 3x 20 min in TBST, then incubated with secondary HRP-conjugated antibodies. Western blots were developed using SuperSignal reagents (Thermo Scientific).

### 4.13. Co-immunoprecipitation followed by western blot

For Co-Ip of GFP fusions, HEK 293 cell lines were transiently transfected with GFP-CEP19 for 24 h after which they were washed with 1× PBS, harvested and frozen at −80°C or lysed immediately (50 mM HEPES pH 8; 100 mM KCl; 2 mM EDTA; 10% glycerol; 0.1% NP-40; 1 mM DTT; protease inhibitors (Roche)) for 30 min on ice. The lysates were then frozen in dry ice for 5 min and then thawed and centrifuged for 30 min at 16 000*g* at 4°C. Protein G Sepharose 4 Fast Flow (P3296 Sigma-Aldrich) were incubated with 2 µg of GFP antibody raised in Goat for 2 h at 4°C and were then washed with lysis buffer. The cleared lysates were then incubated with Sepharose gel (Sigma-Aldrich) for a minimum of 3 h at 4°C. A fraction of the protein extracts (Inputs) were saved before the incubation with the beads. After the incubation, the beads were pelleted and washed with lysis buffer. The samples (Inputs and IPs) were prepared for SDS–PAGE by adding Laemmli buffer and boiling. The proteins were transferred to PVDF membranes (Immobilon-P, Millipore) and probed with antibodies to detect the GFP fusions and endogenous proteins. Primary antibodies used are listed in electronic supplementary material, table S2.

### 4.14. Generation of cell lines

FLAG-CEP19 and GFP-CEP19 constructs were generated via Gateway cloning into pDEST 5′ Triple FLAG pcDNA5-FRT TO or pDEST 5′ GFP pcDNA5-FRT TO (Gateway parental vectors are available from the Gingras laboratory: http://gingraslab.lunenfeld.ca/resources.php?cateName=Reagents). The constructs were validated by sequencing. A total of 293 Flp-In T-REx and Flp_In T-Rex HeLa cells were co-transfected with pOG44 (Flp-recombinase expression vector) and a plasmid containing FLAG-only or FLAG-CEP19, and GFP-CEP19, respectively. Transfections were performed with Lipofectamine 2000 (Invitrogen) according to the manufacturer's instructions. Forty-eight hours post-transfection, cells were selected with Hygromycin B (200 µg ml^−1^).

### 4.15. FLAG affinity purification coupled to mass spectrometry

FLAG-CEP19 (two biological replicates) and FLAG-only 293 cells (four biological replicates) were induced for 24 h with tetracycline (1 µg ml^−1^) to induce the expression of the construct. Cell pellets from two 150 mm plates were lysed in 50 mM HEPES-KOH (pH 8.0), 100 mM KCl, 2 mM EDTA, 0.1% NP-40, and 10% glycerol and affinity-purified with M2-FLAG magnetic beads and on-bead digest as described previously [38]. Spectra were acquired on an LTQ (Thermo Fisher) mass spectrometer placed in line with an Agilent 1100 pump with split flow, essentially as described [38]. To limit carry-over issues, each peptide sample was loaded onto a single-use reversed-phase column with pressure bomb loading. Mascot (v. 2.3) was used for database searching against the human and adenovirus complement of the NCBI RefSeq V53 database. Oxidized methionine and deamidated asparagine and glutamine were considered as variable modifications, and charges +2 and +3 were allowed. Mass tolerance was set at 3 Da for the precursor and 0.6 Da for the fragment ions. Proteins with a minimum ion score of 35 were parsed to the relational module of the ProHits LIMS [39], spectral count matrices were exported for interaction scoring.

### 4.16. Interaction scoring for FLAG affinity purification coupled to mass spectrometry

The five separate biological replicates of negative controls described above were supplemented by six similar controls from the Contaminant Repository for Affinity Purification [40] (controls CC9, CC17, CC22, CC23, CC29 and CC64 were used). SAINTexpress [41] was run through the CRAPome, against a set of five virtual compressed controls: for each potential interactor, the five highest spectral count values across the 11 negative controls (four user controls and six CRAPome controls) was used for modelling to increase the stringency of the scoring, and filter out spurious interactions. SAINTexpress scores for each of the two biological replicate purifications of CEP19 were averaged to a final score (interactors detected in only one purification have an average score less than or equal to 0.5). Fold change was also calculated against the controls.

### 4.17. Lentiviral production and generation of the hTERT RPE-1 GFP-Rab8a stable cell line

The coding sequence of human Rab8a (NM_005370.4) was amplified from human testis cDNAs (total RNA from human testis was purchased from Clontech) and cloned in fusion with GFP in the pcDNA5-FRT/TO-GFP vector. The GFP-Rab8a fusion was subsequently subcloned into the lentiviral vector pHR-SIN-SFFV to generate the pHR-SIN-SFFV-GFP-Rab8a plasmid. For the production of lentiviral particles, HEK293T cells were co-transfected with pHR-SIN-SFFV-GFP-Rab8a and the second-generation packaging (pCMV-dR8.74psPAX2) and envelope (pMD2.G) plasmids using the Lipofectamine 3000 transfection reagent (Invitrogen) according to the manufacturer's instructions. Lentiviral particles in conditioned media from HEK 293T cells, collected at 48 h post-transfection, were used to transduce hTERT RPE-1 cells. GFP-positive cells were cell sorted to establish the final hTERT RPE-1 GFP-Rab8a cell line.

### 4.18. Statistical methods

All *p*-values are from two-tailed unpaired Student *t*-tests. Unless stated, all error bars are s.d. Individual *p*-values, experiment sample numbers and the number of replicates used for statistical testing have been reported in the corresponding figure legends. Unless otherwise stated, we followed this key for asterisk placeholders for *p*-values in the figures: *p < 0.05, **p < 0.01.

## Supplementary Material

Table S1

## Supplementary Material

Table S2

## Supplementary Material

Table S3

## Supplementary Material

Table S4

## Data accessibility

This article has no additional data.
