# Supplementary material for: CEP19 cooperates with FOP and CEP350 to drive early steps in the ciliogenesis programme
Source: Open Biol. 2017 Jun 28;7(6):170114. doi: 10.1098/rsob.170114 (PMC5493781; doi:10.1098/rsob.170114)
Supplement: Table S1 [file rsob170114supp1.docx]

| PreyGene | Quant (SPC or Intensities) | User Controls (SPC or Intensities) | CRAP Controls (SPC) | Avg. SPC | Fold Change | Saint score |
| --- | --- | --- | --- | --- | --- | --- |
| HMGCR | 7\|8 | 0\|0\|0\|0\|0 | 0\|0\|0\|0\|0\|0 | 7.5 | 12.55069 | 1 |
| PPP2R3C | 5\|4 | 0\|0\|0\|0\|0 | 0\|0\|0\|0\|0\|0 | 4.5 | 7.674534 | 1 |
| CEP350 | 19\|13 | 0\|0\|0\|0\|0 | 0\|0\|0\|0\|0\|0 | 16 | 24.34075 | 1 |
| FGFR1OP | 46\|45 | 0\|0\|0\|0\|0 | 0\|0\|0\|0\|0\|0 | 45.5 | 69.94405 | 1 |
| SMARCAD1 | 38\|14 | 0\|0\|0\|0\|0 | 0\|0\|0\|0\|0\|0 | 26 | 36.64978 | 1 |
| SYN2 | 4\|4 | 0\|0\|0\|0\|0 | 0\|0\|0\|0\|0\|0 | 4 | 7.075075 | 1 |
| PCM1 | 16\|9 | 0\|0\|0\|0\|0 | 0\|0\|0\|0\|0\|0 | 12.5 | 18.86513 | 1 |
| RNH1 | 13\|13 | 0\|0\|0\|0\|0 | 0\|0\|0\|0\|0\|0 | 13 | 20.74399 | 1 |
| CALCOCO1 | 4\|1 | 0\|0\|0\|0\|0 | 0\|0\|0\|0\|0\|0 | 2.5 | 4.317145 | 0.77 |
| PAF1 | 4\|0 | 0\|0\|0\|0\|0 | 0\|0\|0\|0\|0\|0 | 2 | 3.397835 | 0.5 |
| ASRGL1 | 3\|0 | 0\|0\|0\|0\|0 | 0\|0\|0\|0\|0\|0 | 1.5 | 2.798377 | 0.49 |
| E2F7 | 2\|1 | 1\|0\|0\|0\|0 | 0\|0\|0\|0\|0\|0 | 1.5 | 2.080994 | 0.48 |
| MFAP3 | 2\|0 | 0\|0\|0\|0\|0 | 0\|0\|0\|0\|0\|0 | 1 | 2.198918 | 0.45 |
| SMC3 | 2\|0 | 0\|0\|0\|0\|0 | 0\|0\|0\|0\|0\|0 | 1 | 2.198918 | 0.45 |
| LYSMD1 | 0\|2 | 0\|0\|0\|0\|0 | 0\|0\|0\|0\|0\|0 | 1 | 2.83862 | 0.45 |
| RABL2A | 2\|0 | 0\|0\|0\|0\|0 | 0\|0\|0\|0\|0\|0 | 1 | 2.198918 | 0.45 |
| CDK1 | 2\|0 | 0\|0\|0\|0\|0 | 0\|0\|0\|0\|0\|0 | 1 | 2.198918 | 0.45 |
| YWHAQ | 5\|0 | 2\|0\|0\|0\|0 | 0\|0\|0\|0\|0\|0 | 2.5 | 2.001787 | 0.45 |
| SDR16C5 | 2\|0 | 0\|0\|0\|0\|0 | 0\|0\|0\|0\|0\|0 | 1 | 2.198918 | 0.45 |
| ANP32A | 2\|0 | 0\|0\|0\|0\|0 | 0\|0\|0\|0\|0\|0 | 1 | 2.198918 | 0.45 |
| ADPRHL2 | 2\|0 | 0\|0\|0\|0\|0 | 0\|0\|0\|0\|0\|0 | 1 | 2.198918 | 0.45 |
| NHLRC2 | 2\|0 | 0\|0\|0\|0\|0 | 0\|0\|0\|0\|0\|0 | 1 | 2.198918 | 0.45 |
| ACTR3 | 2\|0 | 0\|0\|0\|0\|0 | 0\|0\|0\|0\|0\|0 | 1 | 2.198918 | 0.45 |
| ANKHD1 | 2\|0 | 0\|0\|0\|0\|0 | 0\|0\|0\|0\|0\|0 | 1 | 2.198918 | 0.45 |
| NASP | 22\|5 | 1\|3\|4\|2\|12 | 0\|0\|0\|0\|0\|0 | 13.5 | 3.653523 | 0.44 |
| YWHAG | 5\|1 | 0\|0\|3\|0\|0 | 0\|0\|0\|0\|0\|0 | 3 | 3.307963 | 0.44 |
| PYCR2 | 13\|4 | 0\|1\|3\|1\|7 | 0\|0\|0\|0\|0\|0 | 8.5 | 4.06193 | 0.42 |
| PTMA | 4\|1 | 2\|0\|0\|0\|0 | 0\|0\|1\|0\|0\|0 | 2.5 | 2.161964 | 0.42 |
| TUBAL3 | 12\|3 | 0\|0\|0\|0\|7 | 0\|0\|3\|2\|4\|1 | 7.5 | 4.925752 | 0.39 |
| GNL3 | 5\|0 | 0\|0\|0\|0\|0 | 0\|0\|0\|3\|0\|0 | 2.5 | 3.997294 | 0.38 |
| YWHAH | 5\|0 | 0\|0\|3\|0\|0 | 0\|0\|0\|0\|0\|0 | 2.5 | 2.689438 | 0.38 |
| EIF1AX | 6\|0 | 4\|0\|0\|0\|0 | 0\|0\|0\|0\|0\|0 | 3 | 1.535462 | 0.37 |
| IRS4 | 5\|1 | 0\|0\|2\|1\|2 | 0\|0\|0\|1\|0\|0 | 3 | 2.647064 | 0.33 |
| UCHL1 | 3\|0 | 0\|0\|2\|0\|0 | 0\|0\|0\|0\|0\|0 | 1.5 | 2.113265 | 0.32 |
| VPS13D | 0\|3 | 2\|0\|0\|0\|0 | 0\|0\|0\|0\|0\|0 | 1.5 | 1.881917 | 0.32 |
| NME2 | 3\|2 | 0\|0\|3\|0\|0 | 0\|0\|1\|0\|0\|0 | 2.5 | 3.119839 | 0.27 |
| FAM133B | 3\|0 | 0\|4\|0\|0\|0 | 0\|0\|0\|0\|0\|0 | 1.5 | 1.639335 | 0.21 |

**Table S1. AP-MS results of FLAG-CEP19.**

Spectral counts are from two biological replicates. Control counts are derived from 5 negative user control samples (GFP-FLAG) and 6 control samples selected from the CRAPome repository to yield 5 virtual compressed controls (see Materials and Methods). Prey Gene : HUGO gene symbol of each putative prey protein; Spectral counts (SPC) refers to the number of spectra assigned to a given prey polypeptide in a given mass spectrometry (MS) analysis; User Control spectral count: the number of spectra for the indicated prey protein observed in control mass spectrometric analyses; CRAP Controls : the number of spectra for the indicated prey protein observed in the CRAP repository for the selected negative control; Average SPC : Average spectral count for the given prey polypeptide; Fold change : fold change calculation of peptide enrichment against the controls; SAINT: SAINTexpress score [43] assigned to a given polypeptide in any analysis, only preys with corresponding SAINT scores above 0.2 are shown.
